# Unanticipated fake-and-cut maneuvers do not increase knee abduction moments in sport-specific tasks: Implication for ACL injury prevention and risk screening

**DOI:** 10.3389/fspor.2022.983888

**Published:** 2022-11-10

**Authors:** Patrick Mai, Kevin Bill, Katharina Glöckler, Mireia Claramunt-Molet, Julia Bartsch, Mathias Eggerud, Anniken Tidemann Pedersen, Fredrik Sæland, Reidar Bergh Moss, Lasse Mausehund, Steffen Willwacher, Uwe G. Kersting, Ola Eriksrud, Tron Krosshaug

**Affiliations:** ^1^Institute of Biomechanics and Orthopaedics, German Sport University Cologne, Cologne, Germany; ^2^Department of Mechanical and Process Engineering, Offenburg University, Offenburg, Germany; ^3^Digital Health Unit, Eurecat Centre Tecnològic de Catalunya, Barcelona, Spain; ^4^Biomechanical Engineering Lab, Universitat Politècnica de Catalunya, Barcelona, Spain; ^5^Department of Sports Medicine, Oslo Sports Trauma Research Center, Norwegian School of Sport Sciences, Oslo, Norway; ^6^Department of Sport Science, University of Konstanz, Konstanz, Germany; ^7^Department of Physical Performance, Norwegian School of Sports Sciences, Oslo, Norway

**Keywords:** anterior cruciate ligament, joint loading, sports medicine, inverse dynamics, change of direction, cutting, anticipated, handball

## Abstract

Non-contact anterior cruciate ligament injuries typically occur during cutting maneuvers and are associated with high peak knee abduction moments (KAM) within early stance. To screen athletes for injury risk or quantify the efficacy of prevention programs, it may be necessary to design tasks that mimic game situations. Thus, this study compared KAMs and ranking consistency of female handball players in three sport-specific fake-and-cut tasks of increasing complexity. The biomechanics of female handball players (*n* = 51, mean ± SD: 66.9 ± 7.8 kg, 1.74 ± 0.06 m, 19.2 ± 3.4 years) were recorded with a 3D motion capture system and force plates during three standardized fake-and-cut tasks. Task 1 was designed as a simple pre-planned cut, task 2 included catching a ball before a pre-planned cut in front of a static defender, and task 3 was designed as an unanticipated cut with three dynamic defenders involved. Inverse dynamics were used to calculate peak KAM within the first 100 ms of stance. KAM was decomposed into the frontal plane knee joint moment arm and resultant ground reaction force. RANOVAs (α **≤** 0.05) were used to reveal differences in the KAM magnitudes, moment arm, and resultant ground reaction force for the three tasks. Spearman's rank correlations were calculated to test the ranking consistency of the athletes' KAMs. There was a significant task main effect on KAM (*p* = 0.02; $\eta_{p}^{2}$ = 0.13). The KAM in the two complex tasks was significantly higher (task 2: 1.73 Nm/kg; task 3: 1.64 Nm/kg) than the KAM in the simplest task (task 1: 1.52 Nm/kg). The ranking of the peak KAM was consistent regardless of the task complexity. Comparing tasks 1 and 2, an increase in KAM resulted from an increased frontal plane moment arm. Comparing tasks 1 and 3, higher KAM in task 3 resulted from an interplay between both moment arm and the resultant ground reaction force. In contrast to previous studies, unanticipated cutting maneuvers did not produce the highest KAMs. These findings indicate that the players have developed an automated sport-specific cutting technique that is utilized in both pre-planned and unanticipated fake-and-cut tasks.

## Introduction

In team sports, the majority of ACL injuries are non-contact in nature (1, 2), with a subset of these occurring during cutting maneuvers (2). Young female handball players are at greater risk than their male counterparts (1, 3, 4). Athletes participating in ball sports are constantly challenged by interacting with teammates and reacting to opposing players while performing highly dynamic cutting maneuvers, both with and without handling a ball. In handball, video analysis suggests ACL injuries frequently occur during fake-and-cut situations (5, 6). Biomechanical analysis from injury video sequences is limited to a joint kinematic description of injury situations (2, 6). However, joint moments correspond more directly to ligament loading (7), which is currently only measurable in the biomechanics laboratory. Simple game-unspecific lab screening tasks have been suggested for determining the athlete's risk profile, but with poor success (8).

From a biomechanical perspective, the external knee abduction moment (KAM) is likely to contribute to the ACL injury mechanism (2, 6, 9). Therefore, it is essential to reduce KAM through injury prevention training, specifically by targeting fake-and-cut situations. However, it is unclear how different game elements influence knee joint loading. Previous studies have suggested that introducing static defenders, a ball, and an unanticipated change of direction could substantially increase KAM (10). However, an isolated view of such game-specific elements might not reflect the complexity of game scenarios. Therefore, designing laboratory-based tasks that closely mimic game situations more realistically seems necessary to understand joint loading mechanisms (11). Understanding the complex interplay between players and their environment might improve injury risk screening. Hence, the identification of athletes being at high risk for ACL injuries might be significantly improved by implementing game specificity. Yet, it remains unknown if game-specificity increases knee joint loading and if these changes are systematic across players.

Therefore, the purpose of the study was to compare KAM and ranking consistency in female handball players in three sport-specific fake-and-cut tasks of increasing complexity. We hypothesized that the KAM's magnitude increases when athletes face more complex tasks.

## Materials and methods

### Participants

We recruited fifty-one female handball players (mean ± SD: 67.0 ± 7.7 kg, 1.70 ± 0.06 m, 19.2 ± 3.4 years) from various Norwegian handball divisions (Premier, 1st, 2nd, or 3rd division) through personal meetings with coaches and announcements on the Norwegian Handball Association's website. Thirty-five of these 51 players competed in the three highest Norwegian handball divisions. All athletes were at least 16 years of age and played either the back or the wing position. Players in these positions typically perform frequent sidestep cuts during matches. All athletes were pain-free at the time of testing. Seven of the 51 players had recovered from a previous ACL injury. All procedures were followed by the Declaration of Helsinki. The Regional Ethics Committee approved the study before data collection. Informed consent was obtained from all players.

### Experimental setup and protocol

Eighty-two retro-reflective markers of a full-body marker set were attached to the athletes' skin. In detail, lower extremity markers were attached to the following anatomical landmarks: left and right anterior superior iliac spines and posterior superior iliac spines; medial and lateral femoral condyles; medial and lateral malleoli. Furthermore, tracking clusters attached to a rigid shell consisting of four markers were attached to the lateral aspect of the thigh and the shank. Rearfoot markers were placed on the athletes' shoes at the calcaneus' most medial, lateral, and posterior aspects. Forefoot markers were attached to the shoe upper at the head of the first and fifth metatarsal and the head of the first distal phalanx. Following marker attachment, a standardized warm-up routine was carried out, including 5 min of cycling, ten jump squats, seven squats, and seven calf raises.

A three-dimensional marker-based tracking system (24 cameras, Qualisys, Gothenburg, Sweden, 200 Hz) and two floor-embedded force plates (AMTI, Watertown, Massachusetts, USA, 1,000 Hz, 1,200 × 600 mm) sampled the marker trajectories and the ground reaction forces (GRFs) of the athletes during three standardized cutting tasks of different complexities.

For all tasks, the players accelerated for 6 meters and arrived at an angle of ~35° to the long axis of the runway. Athletes performed all tasks at self-selected speeds but were instructed to match game intensity.

**Figure 1:**
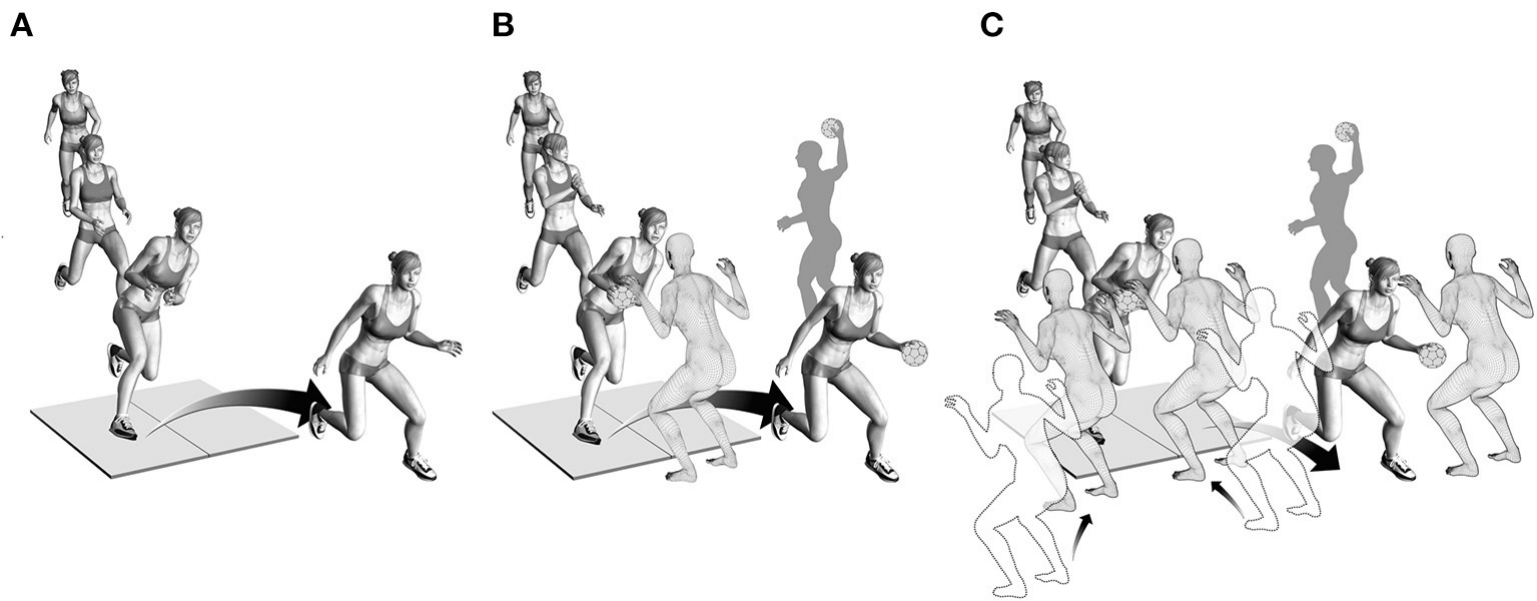
**(A)** Illustration of task 1. Players approached the force plate and performed a pre-planned fake-and-cut maneuver. **(B)** Illustration of task 2. Players caught a ball passed by a teammate while approaching the force plate and performed a pre-planned fake-and-cut in front of a static defender. **(C)** Illustration of task 3. Players caught the ball passed by a teammate while two of the three defenders moved toward the athlete to block one side. This scenario forced the athlete to cut to the unblocked side, resulting in an unanticipated cut.

For task 1, the athletes were instructed to perform a pre-planned fake-and-cut maneuver, similar to what they would do during active gameplay (Figure 1A). No ball or defender was included in this task. Task 2 was performed the same way as task 1, but the athletes would additionally catch a ball passed by a teammate one step before initiating a pre-planned fake and cut in front of a static defender (Figure 1B) (12). For task 3, two defenders were added to either side of the static defender of task 2. The middle defender and one additional randomly selected defender moved toward the athlete at the catch, forcing the athlete to cut away from the moving defenders. This scenario resulted in an unanticipated cut (Figure 1C).

The order of the three tasks was randomized. The players were allowed to familiarize themselves with each task. A minimum of five valid cuts per task was recorded. A cut was considered valid if the foot landed clearly within the boundaries of one of the force plates. For task 2 and task 3, we also tracked the defenders' positions using a marker attached to their back. The cutting leg for tasks 1 and 2 was determined based on playing position and throwing arm, while task 3 was performed on both the left and right leg. However, only the leg determined for tasks 1 and 2 was analyzed for the current study.

### Data analysis

We applied a recursive 4^th^ order low-pass Butterworth filter with a 20 Hz cut-off frequency to the kinematic and kinetic data (13, 14). Knee and ankle joint centers were defined as the midpoints between medial and lateral femoral condyles and malleoli markers. We defined the hip joint centers and pelvis coordinate systems according to Bell et al. and Seidel et al. (15, 16). Segment inertial properties were calculated on anthropometric data from de Leva (17). We determined lower extremity resultant external joint moments with the explicit expression provided by Hof (18) using a rigid body model of the lower extremities, including forefoot, rearfoot, shank, thigh, and pelvis segments. Peak external KAM was normalized to body mass and extracted for the first 100 ms after initial ground contact (IC). IC and toe-off (TO) were defined as time points where the unfiltered vertical GRF component exceeded or fell below 30 N. To test whether changes in the peak KAM are caused by the knee's frontal plane moment arm or the magnitude of the resultant GRF, we analyzed both variables separately. All model calculations were performed using a custom-made Matlab script (R2021a, The Mathworks, Natick, USA). Details of these calculations can be found in previous publications (19–21).

Additionally, we analyzed the horizontal CoM velocity (medio-lateral and anterior-posterior velocity) at initial contact, the distance between attacker and defender at initial contact, and reaction times. We suspected that these variables might explain possible differences in KAMs between the three tasks. For task 3, the athlete's time to decide on a cutting direction and plan the cutting was calculated as the time difference between IC and initiation of the block by the defenders. Initiation of the block by the defenders was defined as the instance when the individual defender's marker velocity reached 0.5 m/s.

### Statistics

To test whether the external peak KAM within the first 100 ms of stance changes with different task complexities, we used a repeated-measures ANOVA. We quantified the effect size using partial eta squared (ηp2). Repeated-measures ANOVAs were additionally carried out for the resultant GRF and the GRF's frontal plane moment arm to the knee joint at the time point of the peak KAM. *Post-hoc* (*p*_*post*−*hoc*_) analysis using Bonferroni-corrected alpha levels was used to identify differences within the three task complexities. Cohen's d effect size for paired samples was calculated to indicate the strength of statistically significant *post-hoc* results. Cohen's d was calculated as the mean differences between two tasks, $\overset{\bar{}}{x_{1}}$ and $\overset{\bar{}}{x_{2}}$, divided by the pooled standard deviation *S*_*pooled*_ (Equation 1). Effect sizes were interpreted as trivial (d = 0–0.19), small (d = 0.20–0.49) medium (d = 0.50–0.79) and large (d ≥ 0.8).


(1) $$\begin{array}{l} d=\frac{\left(\bar{x_{1}}-\;\bar{x_{2}}\right)}{s_{pooled}} \end{array}$$

Additionally, we calculated Spearman's rank correlation coefficient (*r*_s_) for the KAM to assess the athlete's ranking consistency across the three tasks. To test if the timing of the KAM was affected by the task complexity, we used statistical parametric mapping on the time-normalized KAM curves for the first 100 ms after IC (22). All statistical tests were performed using Matlab (The MathWorks, Inc. *MATLAB* 2021a). The significance level was set to α ≤ 0.05.

## Results

### Peak knee abduction moment, frontal plane moment arm, and resultant ground reaction force

**Figure 2:**
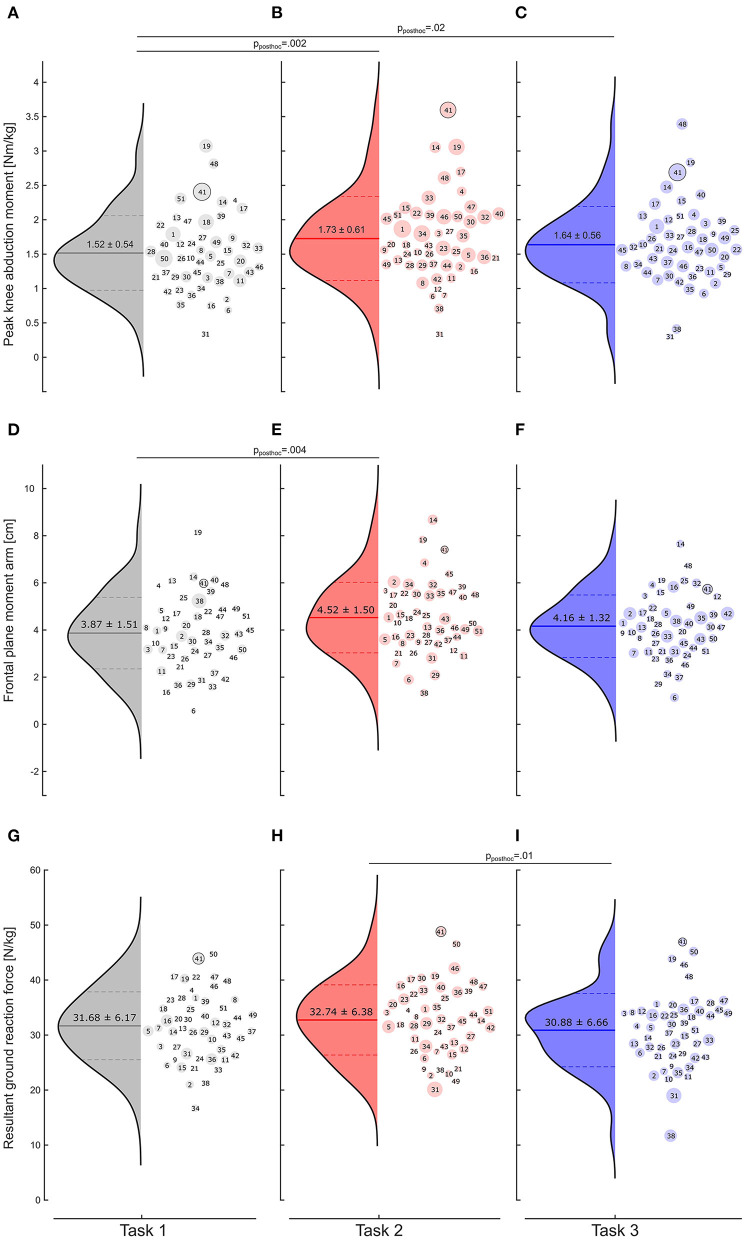
Distribution of the peak knee abduction moment [Nm/kg] within the first 100 ms after initial contact for task 1 **(A)**, task 2 **(B)**, and task 3 **(C)**. Bold lines within the distribution curves represent the average peak knee abduction moment, and dashed lines the standard deviations. The diameters of the dots in the scatters are scaled to the individual standard deviation. The player's number in each scatter helps track the individual rank in each task. **(D–F)** Distribution of the frontal plane moment arms of the knee joint for the three task complexities; **(G–I)** Resultant ground reaction forces and peak knee abduction moments for the three task complexities.

The repeated-measures ANOVA revealed a statistically significant (*p* = 0.02) task effect on the peak KAM within the first 100 ms after IC (Figures 2A–C). Partial eta squared indicated a medium effect size ($\eta_{p}^{2}$ = 0.13). The peak KAM was significantly (*p*_*post*−*hoc*_ = 0.002) higher (+14%) when comparing task 2 (1.73 ± 0.61 Nm/kg) to task 1 (1.52 ± 0.54 Nm/kg), with Cohen's *d* indicating a medium effect size (*d* = 0.50). Comparing the least and most complex tasks, the peak KAM in task 3 (1.64 ± 0.56 Nm/kg) was, on average, 8% higher than in task 1 (*p*_*post*−*hoc*_ = 0.02). Effect size indicate a small effect on KAM magnitudes between the two tasks (*d* = 0.22). On average, the peak KAM was 5% higher in task 2 than in task 3, but no statistical difference could be detected (*p* > 0.05).

Decomposing the peak KAM into the frontal plane moment arm and GRF components, the repeated-measures ANOVA revealed a statistically significant task effect on the moment arm (*p* = 0.002, $\eta_{p}^{2}$ = 0.11) and resultant GRF (*p* = 0.014, $\eta_{p}^{2}$ = 0.08) with both showing medium effect sizes using partial eta squared. The frontal plane moment arm at peak KAM (Figures 2D–F) in task 2 (4.52 ± 1.50 cm) was significantly (*p*_*post*−*hoc*_ = 0.004) longer (+17%) than the moment arm in task 1 (3.87 ± 1.51 cm). Effect size indicates a small effect (*d* = *0.4*7) on the frontal plane moment arm between task 1 and task 2. In task 3, the moment arm at peak KAM (4.16 ± 1.32 cm) was 7% longer than in task 1 and 8% shorter compared to task 2, without any statistical significance (*p* > 0.05). Pairwise comparing the resultant GRF at peak KAM (Figures 2G–I), we found similar magnitudes for task 1 (31.68 ± 6.17 N/kg) and task 2 (32.74 ± 6.38 N/kg). *Post-hoc* tests revealed a statistically significant (*p*_*post*−*hoc*_ = 0.01) 6% lower resultant GRF at peak KAM in task 3 (30.88 ± 6.66 N/kg) than in task 2. A small effect size (*d* = 0.43) on the resultant GRF was observed between the two tasks.

Pairwise comparisons of the peak KAM ranking revealed a very strong (*r*_s_ = 0.80; *p* < 0.001) relationship between task 1 and task 3. On average, the rank change of the athlete's peak KAM was six positions, and the maximal observed rank change was 27. With nine ranks on average and 29 ranks as the most extreme observed rank change, a strong (*r*_s_ = 0.65; *p* < 0.001) relationship in the KAM ranking between task 1 and task 2 was found. When comparing task 2 and task 3, a strong relationship (*r*_s_ = 0.65; *p* < 0.001) was observed. On average, a player changed nine ranks, and 36 ranks were observed as the most extreme rank change.

### Functional knee abduction moment analysis within the first 100 ms of stance

**Figure 3:**
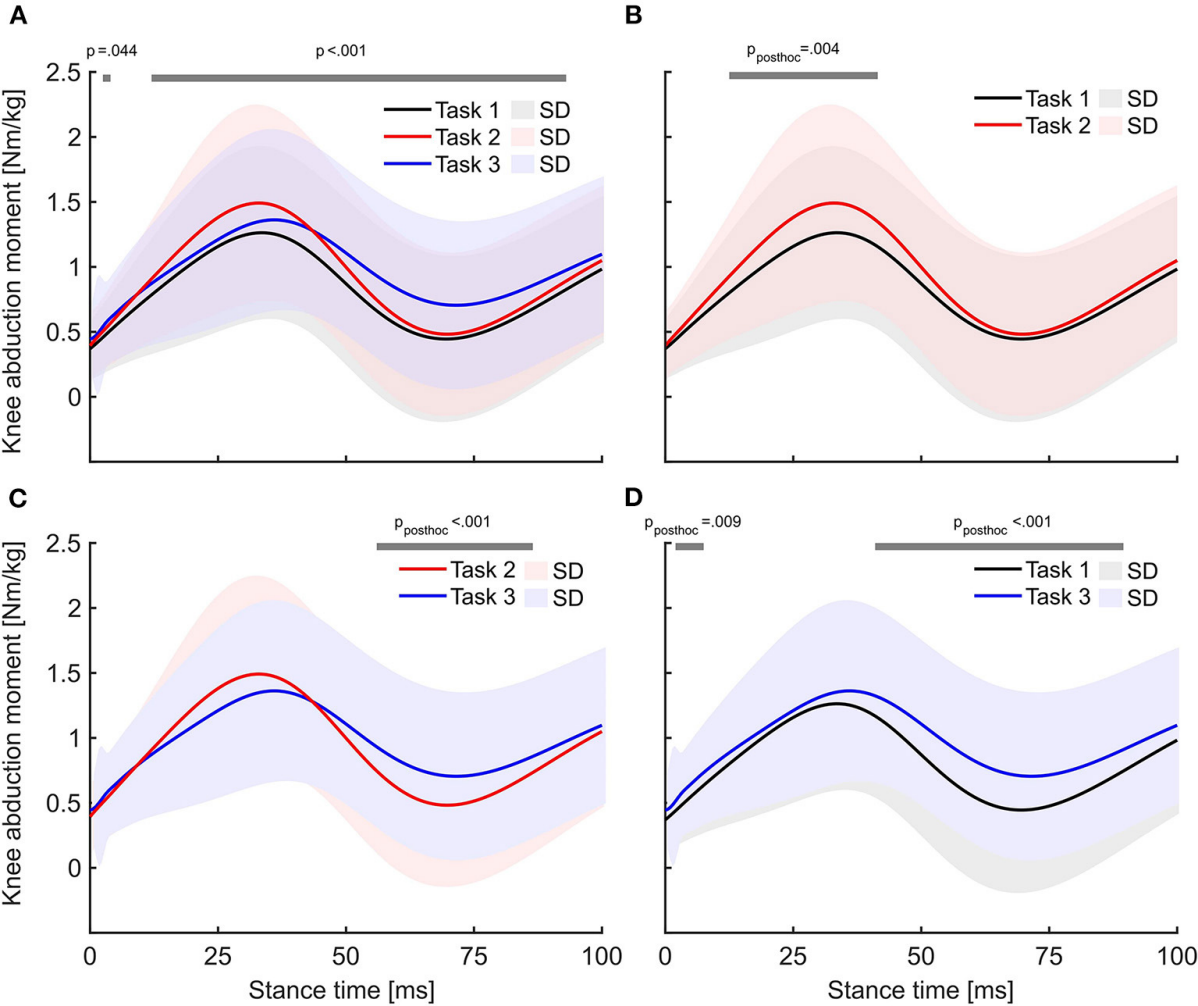
Average external knee abduction moment curves within the first 100 ms of stance. **(A)** Statistical parametric mapping results using repeated-measures ANOVA. The gray lines above the graph indicate significant differences over the respective time interval. **(B–D)** Pairwise comparison of the knee abduction moment curves for each possible task combination, gray lines highlighting significant differences over time using Bonferroni-corrected alpha levels.

Statistical parametric mapping using a repeated-measures ANOVA showed significant differences between the three tasks (Figure 3A). The statistical parametric mapping pairwise comparison revealed significantly (*p*_*post*−*hoc*_ = 0.004) higher KAM for task 2 than for task 1 at 20–45 ms of stance (Figure 3B). When comparing task 2 and task 3, the KAM curves were statistically (*p*_*post*−*hoc*_ <0.001) different within 60–80 ms of stance, with higher KAMs in task 3 (Figure 3C). When comparing the two most extreme tasks, the KAM was elevated in task 3 compared to task 1 (*p*_*post*−*hoc*_ <0.001) within 50–100 ms after IC (Figure 3D). During the first 100 ms of stance, the KAM in task 3 was, on average, higher than in any other task.

### Center of mass kinematics, reaction time, and attacker-defender dynamics

**Figure 4:**
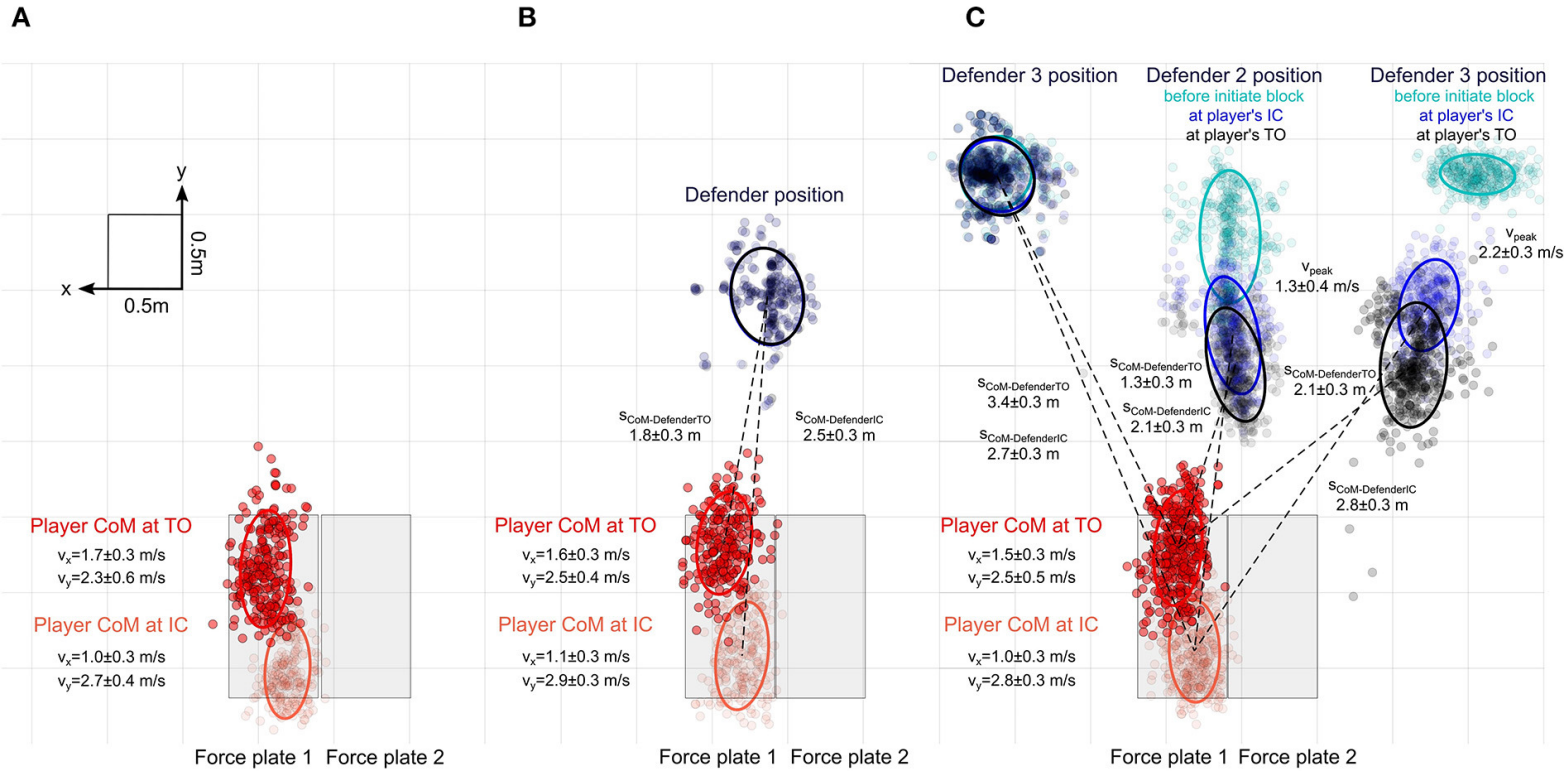
Bird's eye view of the dynamic testing situations. **(A)** Kinematics of the center of mass (CoM) and the point of force application (PoFA) for each cut at initial contact (IC) and toe-off (TO) for task 1. **(B)** Kinematics of the CoM for each cut at IC and at TO, and the position of the static defender for Task 2. **(C)** Kinematics of the CoM for each cut at IC and TO for task 3. Turquoise dots visualize the position of each defender before initiating the block, blue dots represent the defenders' position at the instance of the athletes' IC, and black dots represent the defenders' positions at the athletes' TO. Dashed lines represent the average distance to the respective regions. Ellipses around regions visualize standard deviations.

Since identical movement instructions for the three tasks led to different results in KAMs, we investigated additional variables that might potentially shed light on the causes for these differences. Center of mass (CoM) kinematics of the dynamic test situations were found to be modulated by the task complexity (Figures 4A–C). When comparing the horizontal CoM velocity at IC, the repeated-measures ANOVA revealed a statistically significant (*p* < 0.001; $\eta_{p}^{2}$ = 0.31) effect on the task complexity. *Post-hoc* analysis showed that athletes in task 2 (3.15 ± 0.34 m/s) approached the force plate with a greater horizontal velocity compared to task 1 (2.91 ± 0.35 m/s; *p*_*post*−*hoc*_ <0.001; *d* = 0.32) and task 3 (2.96 ± 0.35 m/s; *p*_*post*−*hoc*_ <0.001; *d* = 0.30). CoM horizontal velocity was not different between task 1 and 3 (*p* = 0.64).

Next, we computed the time the athlete had to react to the defenders in task 3 based on the velocity of the back marker of the dynamic defenders. On average, the athletes had 0.94 ± 0.15 s to plan their motion and initiate the cut to the unblocked side.

Lastly, the repeated-measures ANOVA showed a significant main effect of the task complexity on the cutting angle (*p* < 0.001; $\eta_{p}^{2}$ = 0.27). *Post-hoc* analysis revealed that task 3 (61.5 ± 14.0°) resulted in significantly smaller (*p* < 0.001) cutting angles compared to task 1 (70.8 ± 14.0°). Cohen's *d* indicated a medium effect size (*d* = *0.7*6) on the cutting angle between the two tasks. Statistically significant differences in the cutting angle were additionally observed between task 3 and task 2 (69.2 ± 14.9°), with a medium effect size (*d* = 0.63). No significant differences were found when comparing the cutting angles in tasks 1 and 2.

## Discussion

The study aimed to investigate the effect of match-specific cutting maneuvers with varying task complexities on the peak KAM in female handball players. All three tasks produced substantially higher KAMs than previously reported cutting tasks lacking game specificity (23–25). However, our results are in accordance with results by Kristianslund et al. (12) who assessed KAMs using task 2. In contrast to previous studies (26, 27), the unanticipated cutting maneuver did not generate the highest KAM magnitudes. This finding, combined with the fact that the athlete's KAM ranking was consistent regardless of the task complexity, indicates that the players have developed an automated motor program throughout their careers which is utilized in both pre-planned and unanticipated fake-and-cut tasks. Further, a single complexity might be sufficient for screening purposes. Our findings suggest that their fake-cut technique, including the knee joint loading, is a unique “fingerprint” of each player. Therefore, screening focusing on knee joint loading and cutting technique in faking maneuvers may be an important tool to identify players with increased ACL injury risk.

The potential to modify the fake-cut technique and, hence, reduce knee joint loading is likely higher for young athletes who might not have developed an automated motor program yet (28). Still, previous studies have shown that joint moments are modifiable in teenagers and young adults, e.g., by muscular strength training (29) or altering cutting technique (28) the potential to change the risk profile in all players with high KAM may be substantial.

**Figure 5:**
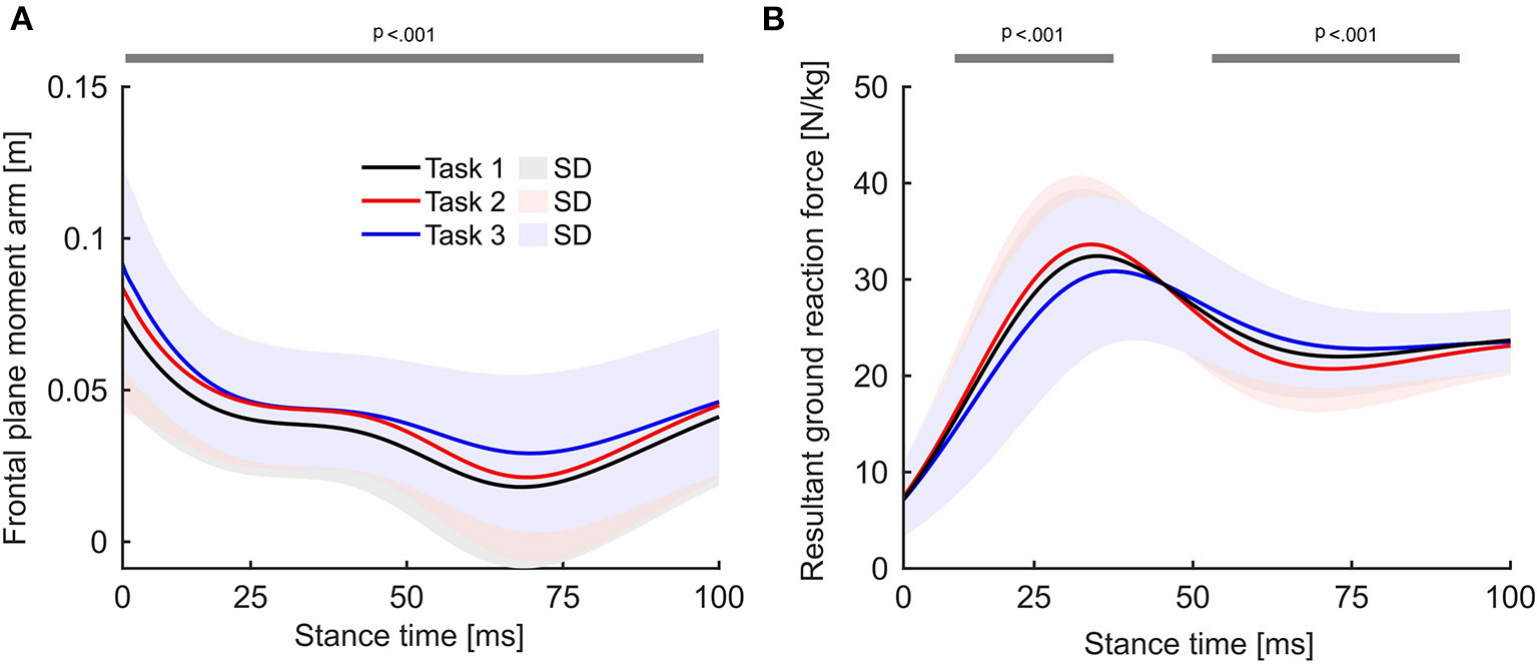
**(A)** Time continuum of the frontal plane moment arm of the knee joint from initial ground contact to 100 ms after ground contact for the three task complexities. **(B)** Time continuum of the resultant ground reaction force for the three task complexities. The gray lines above the graph indicate significant differences over the respective time interval.

In a large-scale cohort study, Kristianslund et al. reported an average peak KAM of 1.64 ± 0.66 Nm/kg when 123 female handball players were screened in a testing scenario equal to task 2 (12). Across all three tasks, the peak KAMs and the timing of the KAM are in agreement with the previously published data (2, 6). Motivated by the experimental design of Kristianslund et al. (12), task 3 in the present study aimed to include the effect of reduced anticipation time on knee joint loading. Kristianslund et al. found that lower knee abduction loads during sidestep cutting result from cutting technique variables, e.g., small cutting angles. A general observation of our data indicates that the athletes performed cuts with smaller cutting angles in task 3 than in tasks 1 and 2. The relationship between knee abduction loading and cutting technique variables might explain why the joint moment in task 3 was not increased compared to task 2. Although the frontal plane moment arm of the knee joint was similar for tasks 2 and 3 at the instance of peak KAM (Figure 5A), the resultant ground reaction force was lower in task 3 than in task 2 (Figure 5B). However, in the present study, it appeared difficult to tightly control for cutting technique variables, e.g., cutting angle and approach speed, due to high variability in solving the given cutting tasks across the athletes. To not interfere with an individual athlete's cutting technique, subjects were not instructed to hit the force plate while cutting. Instead, slight shifts to the starting positions of the dynamic defenders were made to direct the athletes to hit the force plates.

Due to the high number of cuts for the three task complexities, we only analyzed one leg which was selected based on the player's position and the throwing arm. ~2 months after the biomechanical assessment, one athlete sustained a non-contact ACL injury to the left knee (72.3 kg; 1.66 m; 25 yrs, coded as 41 in Figures 2A–C). Even though we only analyzed KAMs for this athlete's right knee joint, the KAM was remarkably high across all three task complexities (task order: 1 – 3 – 2). Moreover, the athlete's ranking was consistent regardless of the task complexity and ranged between the three highest KAMs for the tasks (Figures 2A–C, coded as 41).

Several limitations must be considered when interpreting our results. The first limitation is the uncertainty regarding the lack of anticipation. On average, 0.94 ± 0.15 s elapsed between the dynamic defenders' block initiation and the athletes' contact with the ground in task 3. It is unclear if this time was sufficient to provoke game-like unanticipated cuts. Previous studies (27, 30) using light signals in single-leg landing tasks reported ~0.35–0.65 s between light signal and ground contact. It must be noted that in these studies, subjects could focus on the light signal without any other perturbations. In contrast, subjects in the present study had to react to the defenders and simultaneously look at a teammate passing a ball and catching it before initiating the cut. This might have taken away time for the athletes to react to the defenders, resulting in lower net times to plan their cuts. In the present study, we observed high inter-individual variances in the number of invalid trials due to cuts in the wrong direction, indicating high variance in the time needed to plan and execute the cutting maneuver. We aimed to account for individuality by adjusting the timing of the block initiation to the individual athlete, starting with lower times to react and progressively slightly increasing the time for the athletes until wrong decisions were still present but happened sporadically. It is possible that for some players, the task was too challenging, possibly leading to the slower approach speeds in task 3 relative to task 2 and, in turn, lower KAMs. However, since the defender behavior was adjusted such that the vast majority of cuts were performed to the predetermined side, we believe the task was adequately challenging.

Second, we showed that cutting task complexity affects the KAM. However, it remains unclear which individual automated control strategies regulate the effect of task complexity. For example, subject 1 could keep a constant KAM across the tasks, while other subjects, e.g., subject 40, gradually increased their KAM with increasing task complexity (Figures 2A–C). Besier and co-workers have identified different neuromuscular control strategies for unanticipated compared to anticipated cuts (26). The authors were able to show that in a pre-planned cut, medial leg muscles are activated to support against the externally applied abduction moment. In contrast, a more general co-contraction strategy is adopted in unplanned cuts. While no statistically significant difference in KAM was observed between tasks 2 and 3 in the present study, the muscle activation patterns of the athletes might reveal differences in muscle control strategies. It is unclear how these control strategies might be affected by fatigue, so future studies should investigate the effect of game-specific elements on muscle activation parameters. Further limitations in this study aiming at mimicking game situations might be the lack of spectators, noise, and the psychological pressure associated with these factors.

Lastly, we included athletes with previous ACL injuries. We compared knee kinematics and kinetics of the athletes with a history of ACL injury to the athletes who never sustained an ACL injury. However, we observed no abnormalities in knee joint biomechanics. Moreover, the KAM of the previously injured athletes fell within one standard deviation of the cohort.

Despite these limitations, we could show that a pre-planned fake-and-cut maneuver with additional visual obstacles (i.e., catching a ball and faking a static defender) resulted in similar knee joint loading as a more complex unanticipated fake-and-cut task. Further, the strong to very strong consistencies in the rankings of the athletes' KAMs indicate that these athletes have developed a stable motor program that is used regardless of the task complexity or anticipation level. Therefore, training to internalize cutting technique adaptations such as adopting a forefoot landing or minimizing knee valgus to reduce KAM (12) in a simple pre-planned yet game-specific task might potentially produce carry-over to more complex and unanticipated tasks. These findings have practical implications for researchers and coaches alike, as more time-efficient and less complex screening protocols and training interventions might suffice to identify athletes at risk of injury and to produce adaptations to their neuromuscular control strategies used across sidestep cuts of different complexities.

## Conclusion

In conclusion, we found that unanticipated fake-and-cut maneuvers do not generate higher KAMs than a complex yet pre-planned game-specific task. Interestingly, the correlations between tasks were strong to very strong, indicating that cutting technique and joint loading are “fingerprints” for each player's individual motor program These findings have important clinical implications, suggesting that a sport-specific fake-and-cut maneuver can identify players with high-risk cutting technique.

## Data availability statement

The original contributions presented in the study are included in the article/supplementary material, further inquiries can be directed to the corresponding author.

## Ethics statement

The studies involving human participants were reviewed and approved by the Regional Ethics Committee of the Norwegian School of Sports Science. Written informed consent to participate in this study was provided by the participants' legal guardian/next of kin.

## Author contributions

PM, KB, KG, MC-M, JB, ME, AT, FS, RB, and LM contributed to data acquisition and processing. PM, KB, and SW contributed to the data analysis. TK, UK, and OE contributed to project planning and study design. PM and KB contributed to the draft of the manuscript. UK, SW, OE, and TK helped finalize the manuscript. All authors contributed to the article and approved the submitted version.

## Funding

The Oslo Sports Trauma Research Center has been established at the Norwegian School of Sport Sciences through generous grants from the Royal Norwegian Ministry of Culture, the South-Eastern Norway Regional Health Authority, the International Olympic Committee, the Norwegian Olympic Committee & Confederation of Sport, and Norsk Tipping AS.

## Conflict of interest

The authors declare that the research was conducted in the absence of any commercial or financial relationships that could be construed as a potential conflict of interest.

## Publisher's note

All claims expressed in this article are solely those of the authors and do not necessarily represent those of their affiliated organizations, or those of the publisher, the editors and the reviewers. Any product that may be evaluated in this article, or claim that may be made by its manufacturer, is not guaranteed or endorsed by the publisher.
